# Hysterectomy and Oophorectomy in Reproductive Age: A Cross-Sectional Study from a Tertiary Care Hospital

**DOI:** 10.7759/cureus.8344

**Published:** 2020-05-28

**Authors:** Ruqaiya Shahid, Hina Abbas, Shazia Mumtaz, Fouzia Perveen, Muhammad Furqan Bari, Tazeen Raja, Shaima Memon, Naseem Ahmed, Kartar Dawani

**Affiliations:** 1 Pathology, Dow University of Health Sciences, Dow International Medical College, Karachi, PAK; 2 Hematology, Dow Medical College, Dow University of Health Sciences, Karachi, PAK; 3 Pathology, Dow International Medical College, Dow University of Health Sciences, Karachi, PAK; 4 Gynecology and Obstetrics, Civil Hospital, Karachi, PAK; 5 Pathology, Dow Medical College, Dow University of Health Sciences, Karachi, PAK

**Keywords:** adenomyosis, hysterectomy, leiomyoma, oophorectomy, abnormal uterine bleeding, uterine prolapse

## Abstract

Introduction

Hysterectomy is a common surgical procedure in women, and oophorectomy may also be performed with the hysterectomy. The objective of this study was to identify clinical indications and pathological findings in hysterectomies, performed for gynecological causes, in women of reproductive age (15-49 years) and to determine if oophorectomy or ovarian conservation was performed with the hysterectomy as well as the pathological findings in the ovaries.

Methods

This cross-sectional study was conducted in the department of Pathology at Dow Medical College in Karachi, Pakistan, from September 2017 to December 2018. Data were recorded from the pathology reports of hysterectomy specimens received in the department. Data of hysterectomies performed for gynecological causes in women of reproductive age group were selected and analyzed, using Microsoft Excel (Microsoft, Redmond, Washington) and SPSS version 20 (IBM Corp., Armonk, New York). Data of women more than 49 years and obstetric hysterectomies were excluded.

Results

Three hundred sixty-one hysterectomies were received; 157 of which were hysterectomies performed in women of reproductive age for gynecological reasons. The mean age of the women was 40.37 (± 5.47) years. Abnormal uterine bleeding was the most frequent clinical indication for hysterectomy in 81 (51.59%) women, followed by uterine prolapse in 29 (18.47%) and leiomyoma in 22 (14.01%). Common pathologies in the endometrium were endometritis in 14 (8.92%) and endometrial polyp in nine (5.73%). In the myometrium, leiomyoma was reported in 52 (33.12%) cases, adenomyosis in 37 (23.57%), and both leiomyoma and adenomyosis in 37 (23.57%) women. Uterine prolapse was histologically identified in 38 (24.20%) women. Oophorectomy was performed on 107 (68.15%) women, and out of these, 83 (77.59%) women's ovaries showed either normal histology or functional cysts. Ovarian pathologies reported were endometriosis, serous cystadenomas and oophoritis in five patients each (4.81%), ovarian serous carcinoma in three (2.88%), and mucinous carcinoma in one (0.96%) patient.

Conclusion

Abnormal uterine bleeding was the most common clinical indication for hysterectomy in women of reproductive age. The common pathologies in the hysterectomies were endometritis, endometrial polyp, leiomyoma, adenomyosis, and uterine prolapse. Most of the ovaries removed with the hysterectomy did not show any significant pathology, therefore, further studies in this direction are recommended for confirmation of this finding. Ovarian conservation may be considered in women undergoing hysterectomy for abnormal uterine bleeding or other uterine causes and with no radiological or surgical indication for oophorectomy.

## Introduction

Hysterectomy is a surgery that involves the removal of the uterus of a woman and is one of the most common procedures performed in women of all age groups and is second only to caesarean section procedures in developing countries [1-2]. Oophorectomy is the surgical removal of ovaries and may be performed with hysterectomy [1]. Reproductive age is defined by the World Health Organization (WHO) as ages ranging from 15 to 49 years, during which a woman can become pregnant and is able to bear a child [3].

The most common indication for hysterectomy in reproductive years is abnormal uterine bleeding (AUB), which can be due to benign uterine causes or can have malignant reasons [4]. The International Federation of Gynecology and Obstetrics (FIGO) in 2011 revised the definition of AUB and accepted a new classification system based on the causes of AUB in the reproductive age group while abandoning terms such as menorrhagia and dysfunctional uterine bleeding [4]. An acronym was designed for the causes of AUB: PALM-COEIN (polyp, adenomyosis, leiomyoma, malignancy and hyperplasia, coagulopathy, ovulatory disorders, endometrial, iatrogenic, and not otherwise classified) [4]. In 2018, FIGO modified the nomenclature and included algorithms to guide the clinician in investigating the patient and included sub-classification of leiomyoma based on ultrasound findings [5].

The National Institute for Health and Care Excellence (NICE) recommends hysterectomy as the second line of treatment for bleeding, only after all the appropriate investigations for bleeding have been performed and first-line treatment strategies, which include pharmacological and hormonal treatment and less invasive procedures, have failed [6]. In developed countries, minimally invasive procedures are commonly performed for AUB and include the Levonorgestrel intrauterine system, uterine artery embolization, and endometrial ablation and laparoscopic and robotic hysterectomies, etc. [6-8].

A woman undergoing hysterectomy during her reproductive years bears the brunt of losing her fertility, sexual dysfunction, early menopause, especially if accompanied by oophorectomy, and many psychosocial problems [2]. Hysterectomy is an invasive procedure and is thus also associated with intraoperative and postoperative complications such as injury to the urethra, urinary bladder, ureter, and rectum, excessive bleeding during surgery, wound infection, vault abscess, and urinary tract and lung infections [9-10]. The latest literature from the Cochrane Review suggests that, although hysterectomy has a higher satisfaction rate and improved symptoms of AUB compared to ablation, it is associated with higher postoperative complications, such as sepsis, vault hematoma, wound hematoma, need for blood transfusion and prolonged recovery time [11].

Oophorectomy is electively performed along with hysterectomy, as it might decrease the risk of ovarian cancer; however, there is a lack of consistent scientific evidence for the prophylactic role of oophorectomy, except in cases with known familial genetic mutations for ovarian cancer (BRCA1 and BRCA2) [12]. Bilateral oophorectomy performed in the reproductive years has been causally linked to accelerated aging, increased mortality, and multiple chronic conditions such as dementia, depression, diabetes, hypertension, cancers of all types, arthritis, and chronic kidney and lung diseases [13].

The incidence of hysterectomy is widely variable across the globe. It is estimated to be 20.7/1000 women-years in India [2], which is higher than in Western countries, including the United States (5.1/1000), Australia (4.7/1000), and Germany (3.6/1000) [14-16]. In the West, a decreasing trend of hysterectomy is observed over time, whereas an increasing trend is being reported from Pakistan's neighboring country, India [2, 17-18]. Although no formal rate of hysterectomy has been calculated from Pakistan, a study has reported an increased frequency of hysterectomy in total gynecological admissions, from 7% in 2013 to 17% in 2016 [19]. Therefore, an increasing trend of hysterectomy in developing countries is alarming and efforts should be made to evaluate the clinical indications and pathologies in hysterectomies performed in women of reproductive age, which may be helpful in the allocation of resources for primary health care and prevention of hysterectomy.

This study was conducted primarily to evaluate the clinical indications and pathological findings in hysterectomies performed for gynecological causes in women of reproductive age. Secondarily, this study sought to determine if oophorectomy or ovarian conservation was performed with the hysterectomy and pathological findings in the ovaries.

## Materials and methods

In this cross-sectional study, all reports of hysterectomy specimens received in the department of pathology at Dow Medical College in Karachi, Pakistan, from September 2017 to December 2018 were reviewed. Consecutive, non-probability technique was used for sampling. The reports were retrieved from the computer data system and reviewed with the information recorded by the first and second authors of this study. Microsoft Excel (Microsoft, Redmond, WA) and SPSS version 20 (IBM Corp., Armonk, NY) were used for data collection and analysis. The following variables were recorded: age of the patient, clinical indication for hysterectomy, type of surgery, the status of ovaries (whether bilateral or unilateral oophorectomy was performed with the hysterectomy), and histopathologic findings in the endometrium, myometrium, cervix, fallopian tubes, and ovaries. In keeping with the objective of the study, the data of hysterectomies performed in women of reproductive age (15-49 years), performed for gynecological causes, were selected and analyzed. Data of women older than 49 years and those who underwent obstetric hysterectomy were excluded. Mean was calculated for continuous variable i.e. age. Three age categories of 15-29 years, 30-39 and 40-49 years were made and the frequency of hysterectomy in these age categories was calculated. Frequencies and percentages were calculated for categorical variables i.e. clinical indications and histopathological findings in the endometrium, myometrium, cervix, and ovaries. 

Approval from the Institutional Review Board was taken before the commencement of the study and the reference number is IRB-1087/DUHS/Approval/2018. 

## Results

In the study period of 16 months, 361 hysterectomies were received in the Department of Pathology at Dow Medical College. Of these, 157 (43.49%) were hysterectomies, which were performed in women of reproductive age (15-49 years), and for gynecological causes; 96 (26.59%) were obstetric hysterectomies and 108 (29.92%) were hysterectomies performed in women older than 49 years. The obstetric hysterectomies and gynecological hysterectomies performed in women older than 49 years were excluded. The data of gynecological hysterectomies performed in the reproductive age group were further analyzed. The mean age of women in this group was 40.37 (± 5.47) years. The frequency of hysterectomy of reproductive age was further divided into age categories in Table 1. 

**Table 1:** Distribution of hysterectomy in women of reproductive age (15-49 years).

Age category	Frequency	Percentage
15-29 years	8	5.10%
30-39 years	32	20.38%
40-49 years	117	74.52%
Total	157	100.0%

Abdominal hysterectomy was performed in 128 (81.53%) women and vaginal hysterectomy in 29 (18.47%) women, with 96.55% of vaginal hysterectomies performed in women with the clinical indication of uterine prolapse.

The most common clinical indication for hysterectomy was AUB in 81 (51.59%) women, followed by uterine prolapse in 29 (18.47%), fibroid uterus in 22 (14.01%), and adnexal mass in 13 (8.28%) women (Table 2). 

**Table 2:** Clinical indications for hysterectomy in reproductive age.

Clinical Indication	Frequency	Percentage
Abnormal uterine bleeding	81	51.59%
Uterine prolapse	29	18.47%
Leiomyoma	22	14.01%
Adnexal mass	13	8.28%
Acute abdominal pain	8	5.09%
Not provided	3	1.92%
Infection	1	0.64%
Total	157	100.0%

Analysis of histopathology findings revealed that the endometrium was proliferative in 30 (19.11%) women, secretory in 28 (17.83%), and atrophic in 14 (8.92%) women. The most common pathological finding in the endometrium was endometritis in 14 (8.92%) women, followed by endometrial polyp in nine (5.73%) women (Table 3). 

**Table 3:** Endometrium phases and pathological findings.

Endometrium	Frequency	Percentage
Proliferative	30	19.11%
Secretory	28	17.83%
Non-secretory	24	15.29%
Poorly fixed	21	13.38%
Atrophy	14	8.92%
Endometritis	14	8.92%
Endometrial polyp	9	5.73%
Hormonal effect	8	5.10%
Endometrial hyperplasia	4	2.55%
Disordered proliferative	2	1.27%
Retained products of conception	1	0.64%
Chronic granulomatous inflammation	1	0.64%
Endometrial carcinoma	1	0.64%
Total	157	100.0%

The pathological findings in the myometrium are presented in Table 4. 

**Table 4:** Myometrium and pathological findings.

Myometrium	Frequency	Percentage
Leiomyoma	52	33.12%
Adenomyosis	37	23.57%
Adenomyosis and leiomyoma	37	23.57%
Normal	29	18.47%
Myometritis	2	1.27%
Total	157	100.0%

In the cervix, uterine prolapse was microscopically observed in 38 (24.20%) women.

Ovarian conservation was performed in 50 (31.85%) and oophorectomy was performed in 107 (68.15%) women. Bilateral salpingo-oophorectomy was performed in 97 (61.78%) women, unilateral oophorectomy was performed in 10 (6.37%) women. Out of 107 ovaries, 83 (77.57%) showed either normal histology or functional cysts. The ovarian pathological findings were endometriosis, serous cystadenoma, and oophoritis in five patients each (4.67%). Four (3.74%) cases of ovarian carcinoma were reported, of these three (2.80%) were serous carcinoma and one (0.93%) was mucinous carcinoma (Table 5). 

**Table 5:** Ovaries and pathological findings.

Ovarian Diagnosis	Frequency	Percentage
Functional cysts	46	42.99%
Normal histology	37	34.58%
Endometriosis	5	4.67%
Oophoritis	5	4.67%
Serous cystadenoma	5	4.67%
Mucinous cystadenoma	3	2.80%
Serous carcinoma	3	2.80%
Mucinous carcinoma	1	0.93%
Chronic granulomatous inflammation	1	0.93%
Teratoma (mature)	1	0.93%
Total	107	100.0%

No pathological changes were identified in 93 (87.73%) fallopian tubes. Chronic salpingitis was reported in seven (6.60%) fallopian tubes, hydrosalpinx was identified in two (1.89%), and chronic granulomatous inflammation and endometriosis in one (0.94%) fallopian tube each. 

## Discussion

The results of the study reflected that the principal clinical indications for hysterectomy in women of reproductive age were AUB, uterine prolapse, and leiomyoma. The main pathological findings were leiomyoma and adenomyosis in the myometrium and uterine prolapse in the cervix. The endometrium showed proliferative and secretory patterns in most cases, few cases of endometrial atrophy were noted. Whereas, the foremost endometrial pathologies were endometritis and polyp; endometrial cancer was rare. Oophorectomy was performed in 68.15 % of the women and microscopic examination revealed either normal histology or cysts in more than three-quarters of the ovaries; endometriosis, serous cystadenoma, and oophoritis being the main pathologies observed.

The mean age of women in the reproductive age group was 40.37 (± 5.47) years, whereas, most of the women belonged to the age range of 40-49 years. These findings are comparable to the average age of 41-50 years for women undergoing hysterectomies in developing countries [2, 20]. However, as pathology reports were the only source of information and because of a wide standard deviation in the age of women, a bias of differential misclassification could not be excluded. In the current study, vaginal hysterectomy was performed in 29 (18.47%) women, with 96.55% of vaginal hysterectomies performed in women with the clinical indication of uterine prolapse. Abdominal hysterectomy was performed in 128 (81.53%) women. The proportion of abdominal versus vaginal hysterectomy is similar to other Asian countries such as Nepal (90%) and India (86%) [20-21].

In this study, AUB was reported as the chief clinical indication for hysterectomy in 81 (51.59%) of women. AUB has been verified as the presenting symptom in the majority of women undergoing hysterectomy nationally and internationally [15, 22-23]. A study from India reported AUB to be the presenting complaint in 55.3% of women followed by prolapse in 16.1%, which is similar to our study's findings of AUB in 51.59% and uterine prolapse in 18.47% of women as the clinical indication [22]. According to a study conducted in the United States, one-third of women will be affected by AUB at some point during their life; risk factors are increasing age, premenopausal status, and the presence of leiomyoma and polyps [24]. In women with AUB, hysterectomy is recommended only after all the relevant investigations have been done and conservative treatment options have failed or are refused by the patient [6].

Leiomyoma was the most common tumor diagnosed in the myometrium in 52 (33.12%) women; this finding is comparable to a study from Italy, which showed that leiomyoma was the most common indication for hysterectomy in women less than 55 years old [17]. In the myometrium, other pathologies included adenomyosis in 37 (23.57%) women and adenomyosis in combination with leiomyoma in 37 (23.57%) women. These findings are comparable to studies from Pakistan, India, and Nepal [20, 23, 25]. An Indian study has reported adenomyosis to be the dominant histopathological finding, followed by leiomyoma, with many cases also exhibiting dual pathologies [26].

Uterine prolapse is uncommon at a young age; however, in our study, uterine prolapse was the clinical indication in 29 (18.47%) women and was reported in 38 (24.20%) women, all of whom were older than 25 years. This frequency of prolapse is comparable to a study from Pakistan, which reported prolapse in 24% of hysterectomies, as compared to 37.5% in patients from India and 20% from the United States [23, 25, 27].

In the current study, ovarian cancer was identified in four (3.74%) oophorectomies, whereas 83 (77.57%) ovaries showed either normal histology or functional cysts. An international cohort study on women undergoing simultaneous hysterectomy and oophorectomy for uterine pathologies has reported that ovarian cancer was reported in only 1.5% of bilateral and 1.9% of unilateral oophorectomies removed with the hysterectomy specimen and that benign conditions or normal histology was present in the majority of these oophorectomies [26]. Literature has verified that ovarian conservation for women undergoing hysterectomy for benign causes has long-term survival benefits; for many years post-menopause, the ovaries continue to synthesize testosterone and androstenedione, which is converted to estrogen [27]. Removal of hormonally active ovaries is associated with increased risk of coronary artery disease, hypertension, osteoporosis, fractures, and psychological problems such as depression, Parkinson's disease, schizophrenia, and dementia, as demonstrated in large-cohort studies [28-29]. We, therefore, suggest that ovarian conservation should be considered in women undergoing hysterectomy for AUB, once normal ovaries are confirmed on ultrasound and preoperative examination.

The strength of the current study was that it was a histopathology-based study, which is considered the gold standard in determining the presence and nature of the disease, and was performed in the largest tertiary-care hospital of the province, which receives a heavy patient influx. However, it was limited in that the data were collected from reports in the computer data system and the patients themselves were not contacted for verification. The time duration of the study was short. Multi-center, prospective cohort studies are recommended for verification of findings of this study.

## Conclusions

In this study, AUB was found to be the most common clinical indication for hysterectomy among women of reproductive age. The common pathologies reported were leiomyoma, adenomyosis, and uterine prolapse. Most of the bilateral ovaries removed at the time of hysterectomy had normal histology or functional cysts. Based on this data we suggest that for women of reproductive age undergoing hysterectomy for uterine causes, but without any radiological or surgical indication for oophorectomy, ovarian conservation should be contemplated and opted for to preserve ovarian function.
